# Supplementary figures and images for: Global RNA Expression and DNA Methylation Patterns in Primary Anaplastic Thyroid Cancer
Source: Cancers (Basel). 2020 Mar 13;12(3):680. doi: 10.3390/cancers12030680 (PMC7140095; doi:10.3390/cancers12030680)

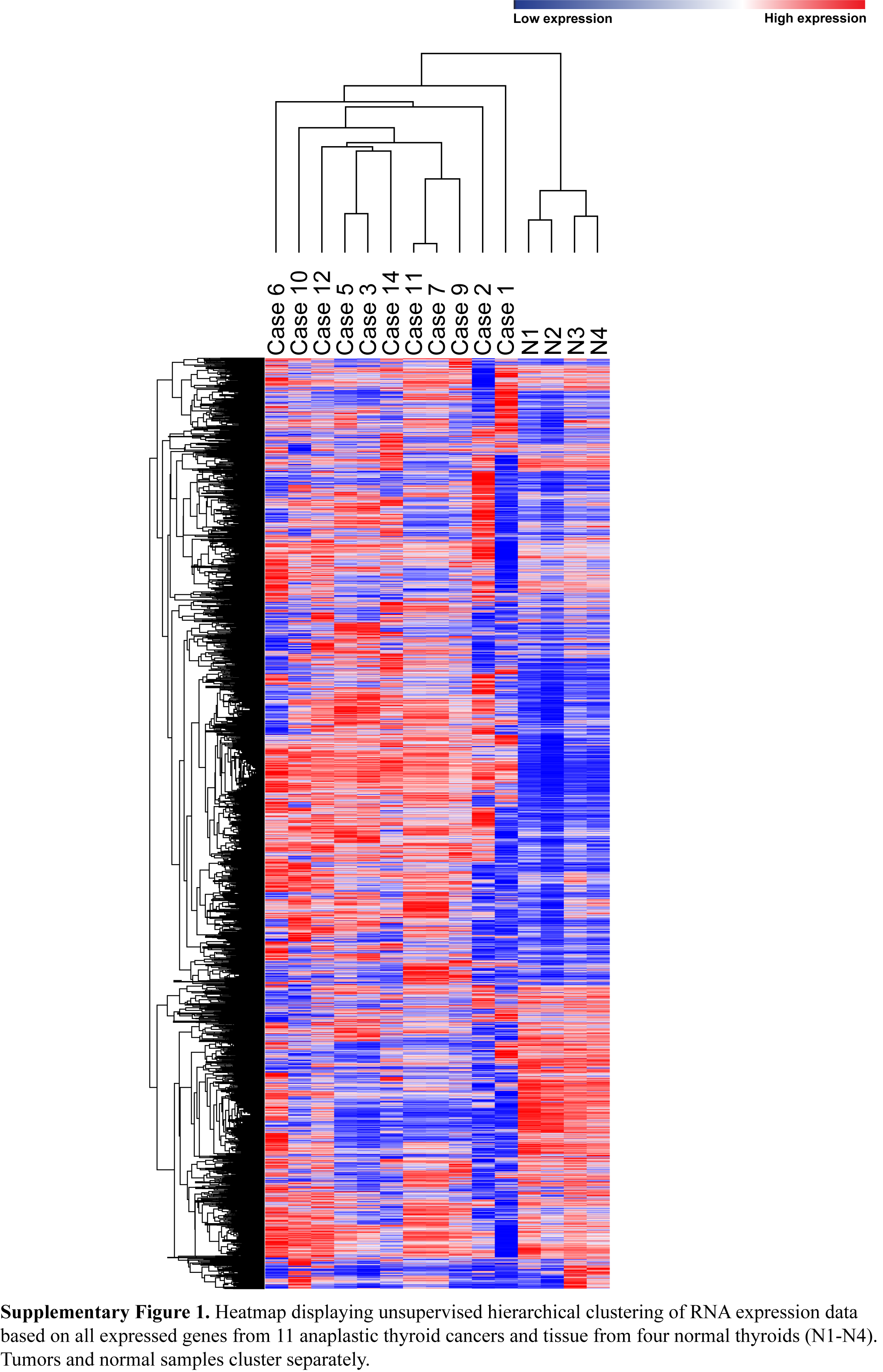

Supplement: Supplementary file 1 [file cancers-12-00680-s001.zip › cancers-724410 - supplementary - proof/Additional Fig 1_v2.tif]

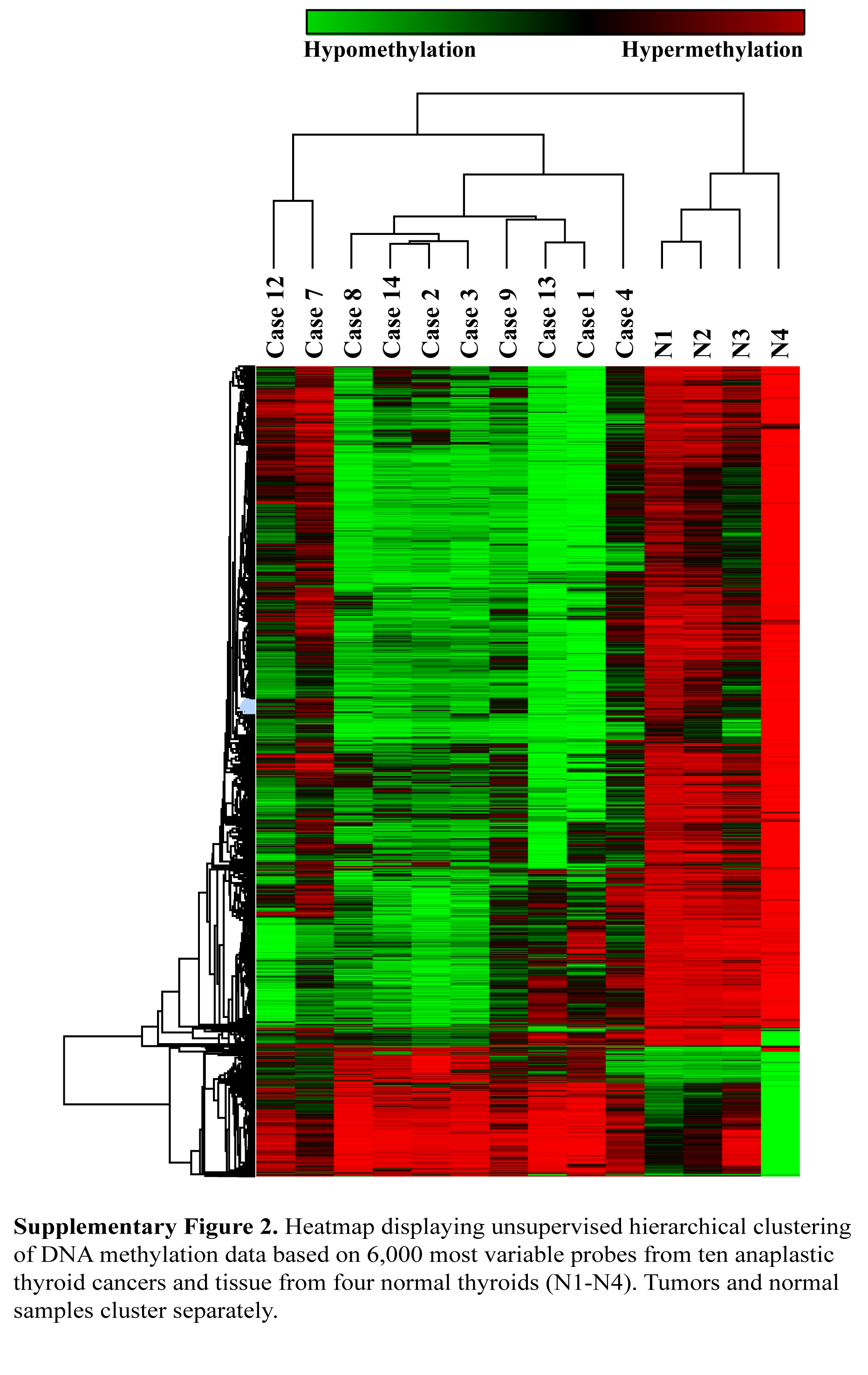

Supplement: Supplementary file 1 [file cancers-12-00680-s001.zip › cancers-724410 - supplementary - proof/Additional Fig 2_v2.tif]
